# Parenteral Nutrition: Current Use, Complications, and Nutrition Delivery in Critically Ill Patients

**DOI:** 10.3390/nu15214665

**Published:** 2023-11-03

**Authors:** Juan Carlos Lopez-Delgado, Teodoro Grau-Carmona, Esther Mor-Marco, Maria Luisa Bordeje-Laguna, Esther Portugal-Rodriguez, Carol Lorencio-Cardenas, Paula Vera-Artazcoz, Laura Macaya-Redin, Beatriz Llorente-Ruiz, Rayden Iglesias-Rodriguez, Diana Monge-Donaire, Juan Francisco Martinez-Carmona, Laura Sanchez-Ales, Angel Sanchez-Miralles, Monica Crespo-Gomez, Cristina Leon-Cinto, Jose Luis Flordelis-Lasierra, Lluis Servia-Goixart, on behalf of the ENPIC Study Group

**Affiliations:** 1 Hospital Clinic, Medical ICU, Clinical Institute of Internal Medicine & Dermatology (ICMiD), C/Villarroel, 170, 08036 Barcelona, Spain; 2 IDIBELL (Biomedical Investigation Institute of Bellvitge), Av. de la Gran Via, 199, 08908 L’Hospitalet de Llobregat, Barcelona, Spain; 3 Intensive Care Department, Hospital 12 de Octubre, Av. de Córdoba s/n, 28041 Madrid, Spain; 4 i+12 (Research Institute Hospital 12 de Octubre), Av. de Córdoba s/n, 28041 Madrid, Spain; 5 Intensive Care Department, Hospital Universitario Germans Trias i Pujol, Carretera de Canyet, s/n, 08916 Badalona, Barcelona, Spain; 6 Intensive Care Department, Hospital Clínico Universitario de Valladolid, Av. Ramón y Cajal, 3, 47003 Valladolid, Spain; 7 Intensive Care Department, Hospital Universitari Josep Trueta, Av. de França, s/n, 17007 Girona, Spain; 8 Intensive Care Department, Hospital de la Santa Creu i Sant Pau, C/Sant Quintí, 89, 08041 Barcelona, Spain; 9 Intensive Care Department, Complejo Hospitalario de Navarra, C/Irunlarrea, E, 31008 Pamplona, Navarra, Spain; 10 Intensive Care Department, Hospital Universitario Príncipe de Asturias, Av. Principal de la Universidad, s/n, 28805 Alcalá de Henares, Madrid, Spain; 11 Intensive Care Department, Hospital General de Granollers, C/Francesc Ribas, s/n, 08402 Granollers, Barcelona, Spain; 12 Intensive Care Department, Hospital Virgen de la Concha, Av. Requejo, 35, 49022 Zamora, Spain; 13 Intensive Care Department, Hospital Regional Universitario Carlos Haya, Av. de Carlos Haya, 84, 29010 Málaga, Spain; 14 Intensive Care Department, Hospital de Terrassa, C/Torrebonica, s/n, 08227 Terrassa, Barcelona, Spain; 15 Intensive Care Department, Hospital Universitari Sant Joan d’Alacant, N-332, s/n, 03550 Sant Joan d’Alacant, Alicante, Spain; 16 Intensive Care Department, Hospital Doctor Peset, Av. Gaspar Aguilar, 90, 46017 Valecia, Spain; 17 Intensive Care Department, Hospital Royo Villanova, Av. San Gregorio, s/n, 50015 Zaragoza, Spain; 18 Intensive Care Department, Hospital Universitari Arnau de Vilanova, Av. Alcalde Rovira Roure, 80, 25198 Lleida, Spain; 19 IRBLLeida (Lleida Biomedical Research Institute’s Dr. Pifarré Foundation), Av. Alcalde Rovira Roure, 80, 25198 Lleida, Spain

**Keywords:** parenteral nutrition, enteral nutrition, complementary parenteral nutrition, critically ill patients

## Abstract

Background: Parenteral nutrition (PN) is needed to avoid the development of malnutrition when enteral nutrition (EN) is not possible. Our main aim was to assess the current use, complications, and nutrition delivery associated with PN administration in adult critically ill patients, especially when used early and as the initial route. We also assessed the differences between patients who received only PN and those in whom EN was initiated after PN (PN-EN). Methods: A multicenter (*n* = 37) prospective observational study was performed. Patient clinical characteristics, outcomes, and nutrition-related variables were recorded. Statistical differences between subgroups were analyzed accordingly. Results: From the entire population (*n* = 629), 186 (29.6%) patients received PN as initial nutrition therapy. Of these, 74 patients (11.7%) also received EN during their ICU stay (i.e., PN-EN subgroup). PN was administered early (<48 h) in the majority of patients (75.3%; *n* = 140) and the mean caloric (19.94 ± 6.72 Kcal/kg/day) and protein (1.01 ± 0.41 g/kg/day) delivery was similar to other contemporary studies. PN showed similar nutritional delivery when compared with the enteral route. No significant complications were associated with the use of PN. Thirty-two patients (43.3%) presented with EN-related complications in the PN-EN subgroup but received a higher mean protein delivery (0.95 ± 0.43 vs 1.17 ± 0.36 g/kg/day; *p* = 0.03) compared with PN alone. Once adjusted for confounding factors, patients who received PN alone had a lower mean protein intake (hazard ratio (HR): 0.29; 95% confidence interval (CI): 0.18–0.47; *p* = 0.001), shorter ICU stay (HR: 0.96; 95% CI: 0.91–0.99; *p* = 0.008), and fewer days on mechanical ventilation (HR: 0.85; 95% CI: 0.81–0.89; *p* = 0.001) compared with the PN-EN subgroup. Conclusion: The parenteral route may be safe, even when administered early, and may provide adequate nutrition delivery. Additional EN, when possible, may optimize protein requirements, especially in more severe patients who received initial PN and are expected to have longer ICU stays. NCT Registry: 03634943.

## 1. Introduction

Enteral nutrition (EN) is usually the main route for providing nutrition therapy in patients admitted to the intensive care unit (ICU), however, parenteral nutrition (PN) may be needed to avoid the development of malnutrition when EN is contraindicated or unfeasible. It has been extensively recognized that appropriate nutritional support is indispensable to critically ill patients; nevertheless, a systematic review of malnutrition diagnosed by validated nutrition assessment tools revealed a strikingly high prevalence of malnutrition in ICU patients (ranging from 38% to 78%), which is associated with poor outcomes including increased morbidity, mortality, and hospital-related costs [1]. On the other hand, recommendations for nutrition in critically ill patients have been published in different clinical practice guidelines [2,3,4,5] but nutrition targets are frequently difficult to achieve in routine bedside practice [6,7].

EN is more physiological, with various non-nutritional benefits (e.g., maintenance of structural and functional gut integrity, preservation of gut microbiome) but also disadvantages related to potential lower nutritional adequacy, particularly in the acute disease phase and in the presence of gastrointestinal dysfunction [8]. In contrast, total PN may better secure the intended nutritional intake, but it may be associated with more infectious complications and longer ICU stays [9]. However, in a large randomized controlled trial, involving 2388 patients, which evaluated the effect of the nutrition route on the outcomes of critically ill adult patients, neither a significant difference in 30-day mortality nor in the number of treated infectious complications was found between patients receiving total PN or EN [10]. Also, in a previous nationwide analysis of nutritional practices in critically ill patients admitted to different ICUs, differences in 28-day mortality between the groups of patients with EN or total PN were not observed [11].

The present research was conducted to provide further analysis of the use of PN in adult critically ill patients, especially when used early and as the initial route. The objectives of the study were to assess the current use and complications associated with PN, to evaluate nutrition delivery (i.e., caloric and protein) with the use of PN, and to assess the differences in nutrition therapy between patients who received PN only (i.e., PN subgroup) and those in whom EN was initiated after PN (i.e., PN-EN subgroup).

## 2. Materials and Methods

### 2.1. Design and Setting

This was a nationwide prospective observational and multicenter study conducted in 37 Spanish ICUs. The results of the present research correspond to a planned analysis of the insights obtained from the use of PN in the ENPIC study. All consecutive patients admitted from 1 April to 31 July 2018 were included in the study. Patients aged 18 years or older admitted to the participating ICUs and requiring artificial nutrition support for more than 48 h, with an expected stay of more than 72 h, were eligible. Only patients who required initial PN on ICU admission were included in the analysis of the present study. We also included those patients in whom EN was initiated after initial PN. Patients admitted to the ICU for postoperative recovery from a surgical procedure and/or ICU monitoring not requiring specific treatment for organ support (e.g., vasopressors or non-invasive mechanical ventilation) were excluded from the study. Patients who were able to feed orally were also excluded. Eligible ICUs were required to follow a nutritional protocol consistent with current clinical guidelines or to involve medical staff specialized in artificial nutrition therapy [6].

The study protocol was approved by the Clinical Research Ethics Committee of Hospital Universitari de Bellvitge (code PR401/17), which is a central institutional review board. The requirement of informed consent was waived because of the observational design of the study and data being collected from an anonymous centralized database. Patients included in the present study were obtained from the Evaluation of Nutritional Practices in the Critical Care registry (ENPIC Study; ClinicalTrials.gov Identifier: NCT 03634943).

### 2.2. Data Collection and Study Endpoints

The data collected for each patient were as follows: demographics; body mass index (BMI); comorbidities (e.g., hypertension, diabetes mellitus, chronic obstructive pulmonary disease {COPD}, acute myocardial infarction, chronic renal failure, immunosuppression, and active cancer); type of patient (medical, trauma, or surgery); Acute Physiology and Chronic Health Evaluation (APACHE) II score; Simplified Acute Physiology Score (SAPS) II score; Sequential Organ Failure Assessment (SOFA) score on ICU admission; nutrition status using the Subjective Global Assessment (SGA) and modified Nutrition Risk in the Critically Il (mNUTRIC) score; details of nutritional therapy, including time of initiation of PN, early PN within the first 48 h after ICU admission, and energy and protein intake during the entire administration of nutrition therapy or at least for the first 14 days; laboratory data (lipid profile, liver parameters, and blood proteins) at admission, day 3, day 7, and at ICU discharge; and outcomes during ICU stay, which included the need for invasive mechanical ventilation and days on invasive mechanical ventilation, use of vasoactive drug support, need for renal replacement therapy (RRT), respiratory tract infection, catheter-related bloodstream infection, length of stay in the ICU and hospital, and 28-day mortality rate. In the database, we evaluated and registered mechanical complications (e.g., thrombosis, pneumothorax, etc.) related to catheter insertion and maintenance, PN-related catheter infections, and laboratory-related abnormalities (e.g., cholestasis, hypertriglyceridemia, etc.) with the use of PN. Mechanical, infectious, and metabolic complications related to PN use were screened daily during ICU admission via a physical exploration (e.g., examination of the percutaneous entry sites of central venous catheters for the presence of local inflammation and purulence), chest X-ray (e.g., screening for the presence of pneumothorax or pleural effusion), and echography if necessary (e.g., to detect venous thrombosis, veno-arterial fistulae, etc.). Investigators systematically performed assessments in order to detect and diagnose these complications related to the use of PN, which are described in detail in the Supplementary Material (Table S1). Alterations in laboratory data were scrutinized and analyzed to show association during PN administration. Liver dysfunction was defined according to the presence of the following criteria: (a) cholestasis: alkaline phosphatase > 280 IU/L, gamma-glutamyl-transferase > 50 IU/L, or bilirubin > 1.2 mg/dL; (b) liver necrosis: aspartate aminotransferase > 40 IU/L, alanine aminotransferase > 42 IU/L, or INR > 1.4; and (c) mixed pattern: cholestasis and liver necrosis [12]. To evaluate nutrition delivery with the use of PN, we compared the caloric and protein delivery of those who received only EN with those who only received PN in our patient cohort. We also analyzed the data from those patients who received PN after initial EN (EN-PN subgroup) for descriptive purposes.

When comparing patients receiving PN as their initial nutrition therapy (PN subgroup) and those who also received complementary EN after initial PN (PN-EN subgroup), the study endpoints were the differences in outcomes during their ICU stay (i.e., mechanical ventilation, vasoactive drug support, RRT, respiratory and catheter-related infections, PN-related complications, length of ICU and hospital stay, and 28-day mortality), including laboratory data (e.g., presence of liver dysfunction). In the PN-EN subgroup, EN-related complications were also registered. The presence of a high gastric residual volume (GRV) was defined as an aspirated volume from the stomach of >500 mL via a nasogastric tube following EN administration. Aspirations were performed at the discretion of each clinician based on the observational nature of the present study.

### 2.3. Statistical Analysis

Categorical variables are expressed as frequencies and percentages, and continuous variables as means and standard deviations (SDs). The distribution of categorical variables between study groups was compared with the chi-square test and the distribution of continuous variables with the two-sample *t*-test or the Mann–Whitney U test. Statistical significance was set at *p* < 0.05 (two-tailed).

Subsequent multivariate analysis was conducted using an adjusted multiple stepwise Cox regression analysis to add a time perspective. Variables were included in the initial model if they had a *p*-value < 0.2 and were deemed suitable by the investigators based on careful consideration of confounding. Investigators selected variables based on current knowledge and the literature perspective [13]. We used the change-in-estimates criterion and backward deletion with a 10% cut-off to eliminate variables from the final model. To avoid destabilizing the multivariate analyses, we tested for interactions between all variables introduced. We then adjusted for age, patient type (e.g., medical, surgical, or trauma), illness severity (e.g., APACHE score), length of nutritional therapy, and data presenting significant differences in the baseline characteristics between both subgroups. This helped avoid confounders and the influence of illness severity when analyzing outcomes. Hazard ratios (HRs) and 95% confidence intervals (CIs) were estimated. Statistical analysis was conducted using PASW statistics 20.0 (SPSS Inc., Chicago, IL, USA).

In all cases, the Kolmogorov–Smirnov and D’Agostino–Pearson omnibus normality tests were used to check the normal distribution of our population and assess the goodness-of-fit of the final regression models.

## 3. Results

### 3.1. Population Included in the Study

**Figure 1:**
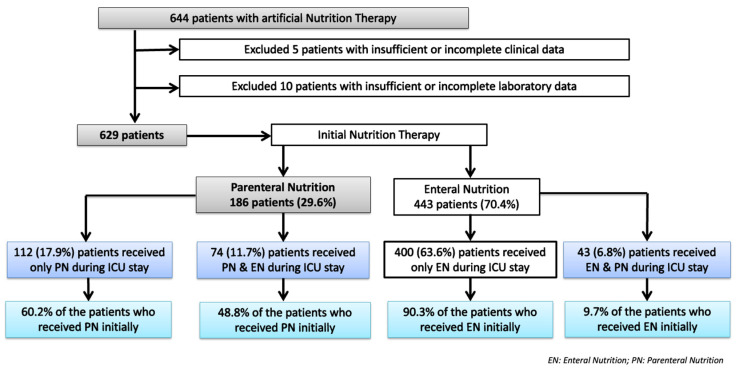
Study flow chart of patients included in the study.

A total of 644 ICU patients received artificial nutrition therapy during the study period; however, 15 patients were excluded due to insufficient or incomplete clinical (*n* = 5) or laboratory data (*n* = 15). Therefore, the remaining 629 were included in the study, 186 (29.6%) of whom received PN and 443 (70.4%) of whom received EN as the initial delivery route of nutrition therapy. Of the 186 patients treated with PN, 74 also received EN during ICU admission (PN-EN subgroup). These 74 patients from the PN-EN subgroup accounted for 39.8% of those who received PN and 11.8% of all patients included in the study. Of the 443 patients treated with initial EN, 400 received only EN, and 43 needed PN (EN-PN subgroup). A total of 36.4% of the patients (*n* = 229) received PN at some point during their ICU admission. The patient population flow chart is shown in Figure 1.

### 3.2. Current Use and Complications Associated with PN

PN was indicated by the attending intensivist due to the impossibility of initial oral feeding or EN. It is important to remark that a daily evaluation to replace PN with EN was performed on each patient [2].

General characteristics, nutritional support, and outcomes of the study population are shown in Table 1. Patients were admitted due to medical and major surgical diseases, and more than half of the patients presented with malnutrition (59.1%; *n* = 110) based on the SGA scale. Additionally, the main reasons reported for administering PN were the following: paralytic ileus after major surgery (44.1%; *n* = 82), bowel rest (e.g., acute pancreatitis, Crohn’s disease) (17.2%; *n* = 32), bowel fistula (14.5%; *n* = 27), occlusion or stenosis of the digestive tract (8.6%; *n* = 16), and short bowel (3.2%; *n* = 6). Other patients presented further contraindications for the enteral route (*n* = 18) based on the severity of the diagnosis (e.g., hemodynamic instability, active digestive bleeding) and in only two patients, a nasogastric tube could not be inserted.

PN was given for a mean duration of 13 days (range 4–16 days). Only 29 (15.6%) patients received a short course of nutrition therapy (>48 to 72 h), 46 (24.7%) received an intermediate course (>72 h to <7 days), and 111 (59.7%) received a long course (≥7 days). In the majority of patients, PN was administered via a central catheter (89.2%; *n* = 166) or a central catheter inserted through a peripheral vein (9.1%; *n* = 17), whereas only three patients received PN via a peripheral line (1.6%; *n* = 3).

The majority of patients received early PN (i.e., during the first 48 h of ICU admission). No major complications (e.g., mechanical complications, such as pneumothorax, thrombosis, etc.) affecting the outcomes were found to be associated with the use of PN, and 8.1% (*n* = 15) of the patients suffered catheter-related infections, which were not associated with the use of PN per se either. Regarding laboratory data, we found a low incidence of lipid profile alterations, such as hypertriglyceridemia (i.e., >350 mg/dL), in 8.6% (*n* = 16) of patients; however, liver dysfunction was frequent (40.3%; *n* = 75) and most had cholestasis (25.8%; *n* = 48), whereas a low proportion had hepatic necrosis (9.1%; *n* = 17) and mixed patterns of liver dysfunction (5.4%; *n* = 10). Nevertheless, in most patients, liver dysfunction was unrelated to the use of PN as it was present from the start of ICU admission in almost all patients (*n* = 72). Only three patients developed hypertriglyceridemia during PN use. Finally, a progressive increase in prealbumin levels was seen with nutrition therapy in the entire population (*p* = 0.01).

### 3.3. Assessment of Nutrition Delivery

In our study, all patients with initial PN received a mean caloric and protein delivery superior to 70% of requirements based on national and international guideline recommendations (i.e., 20 Kcal·Kg^−1^·day^−1^ and 1.2–1.3 g of protein·Kg^−1^·day^−1^) [2,3,4,5]. When comparing the caloric and protein delivery of those who received only EN (*n* = 400) with those who received only PN (*n* = 112), to assess nutritional therapy delivery with PN, we found a similar delivery with both routes, with a trend towards better delivery with PN (Table 2 and Table 3). Indeed, multivariate analysis, adjusted for confounding factors, revealed a trend toward higher mean caloric delivery with PN (HR: 1.20; 95% CI: 1.13–1.27; *p* < 0.001). Patients who received only EN were more likely to be medical patients (HR: 5.56; 95% CI: 3.01–10.23; *p* = 0.001), presented neoplasia as a comorbid condition (HR: 0.42; 95% CI: 0.22–0.81; *p* = 0.01), and had higher invasive mechanical ventilation needs (HR: 6.95; 95% CI: 1.12–7.76; *p* < 0.001). Of the patients with only EN, 26% (*n* = 104) had EN-related complications (high GRV: 11.5% (*n* = 46); diarrhea: 8.7% (*n* = 35); vomiting: 1.2% (*n* = 5); and mesenteric ischemia: 0.7% (*n* = 3)). The EN-PN subgroup (*n* = 43) shared similar characteristics with only the EN subgroup (Table 2), and it did not show other novel findings when compared with other patients receiving PN (i.e., only PN and the PN-EN subgroup), even regarding mean caloric and protein delivery (Table S2). However, the EN-PN subgroup showed higher EN-related complications (60.5% (*n* = 26) suffered from any type of EN-related complications; high GRV: 37.2% (*n* = 16); diarrhea: 18.6% (*n* = 8); and mesenteric ischemia: 9.3% (*n* = 4)).

### 3.4. Differences between the PN and PN-EN Subgroups

**Figure 2:**
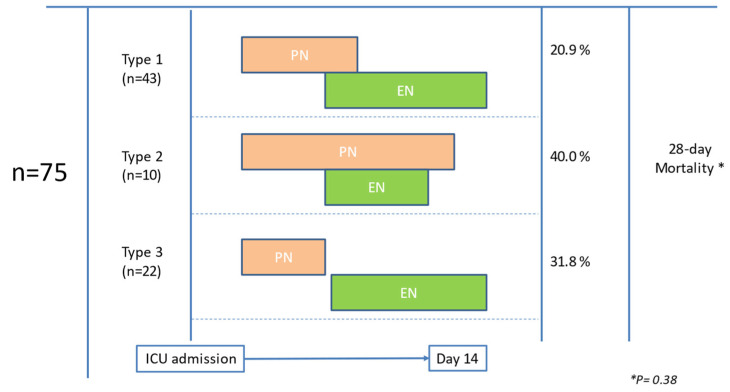
Patterns of administration of parenteral (PN) and enteral nutrition (EN) in patients who initially received PN.

The PN-EN subgroup of 74 patients had different administration patterns (see Figure 2), including initiation of EN after discontinuation of PN (Type 2; *n* = 22) or progressive switching from PN to EN (Type 1; *n* = 43). In a small number of PN-EN subgroup patients (Type 3; *n* = 10), however, EN was stopped after initiation and the switch from PN to EN was not feasible, the main reason being the impossibility of providing the appropriate nutritional needs due to the presence of a high GRV. These ten patients all received prokinetics (i.e., combined Metoclopramide and Erythromycin), to try to improve the high GRV during EN administration, and EN was administered simultaneously with PN for a mean of 5.5 days (5 to 8 days). However, EN had to be completely stopped because the prokinetic treatment was unresponsive and the GRVs remained high. Differences in the use of PN-EN therapy within the different administration patterns were not associated with significant differences in the 28-day mortality rate. A more precise analysis of the different patterns of PN-EN administration was not performed in this subgroup due to the lack of a sufficiently large number of patients in each group, impeding accurate statistical analysis. EN was initiated in the PN-EN subgroup during the first week of ICU admission in all cases (between days 4 and 7).

Differences between the general characteristics, nutritional support, and outcomes of these subgroups are shown in Table 1. Patients in the PN-EN subgroup showed statistically significant differences compared with the PN subgroup; they were more frequently medical patients with higher organ failure (i.e., higher SOFA scores) on ICU admission, and represented a subgroup with greater severity and need for organ support, which is seen via the higher requirements of mechanical ventilation, days on mechanical ventilation, use of vasoactive drugs, and longer duration of ICU and hospital stays. Moreover, a higher percentage of patients in the PN-EN group received nutrition therapy for at least 7 days (86.5%) compared to patients treated with PN only (42.0%) (*p* < 0.001), which may reflect longer ICU stays.

Patients from the PN-EN group received a higher mean caloric and protein delivery during the first week and higher mean protein delivery during the second week of ICU admission (Table 4). More than one-third of patients in the PN-EN group had EN-related complications (43.3%; *n* = 32). The most frequent complication was a high GRV (25.7%; *n* = 19), followed by diarrhea (10.8%; *n* = 8). Vomiting (2.7%; *n* = 2) and aspiration (1.3%; *n* = 1) were both infrequent complications, and mesenteric ischemia was diagnosed in only one patient.

Multivariate analysis, also adjusted for confounding factors, revealed differences among subgroups: patients in the PN group received a lower mean protein intake during nutrition therapy during ICU admission, had a shorter ICU stay, and less time on mechanical ventilation compared with those in the PN-EN group (Table 5).

Regarding laboratory data, there was a trend toward higher triglyceride levels in the lipid profiles of the PN-EN group (without statistical significance) and no differences in liver dysfunction between the PN and PN-EN groups were found. The other laboratory parameters at ICU admission, at days 3 and 7, and at the time of discharge were quite similar between the PN and PN-EN subgroups. More reliable results regarding the laboratory analysis are shown in Table 6, whereas the complete results are shown in the Supplementary Material (Table S3).

## 4. Discussion

The present observational research focused on the analysis of the nutrition practices associated with the use of PN, especially when used early and as the initial route. In our population, PN was required by one-third of the patients. We show that the use of PN as the initial route for nutrition therapy, even when initiated early, may be not associated with major complications in adult critical care patients and may help provide adequate nutrition delivery during the entire ICU admission. Most importantly, our results suggest that nutrition therapy in those patients who received initial PN and are expected to have longer ICU stays (i.e., those with a higher degree of organ failure on ICU admission and higher needs for invasive mechanical ventilation) may potentially benefit from considering EN as complementary to PN or progressively switching from PN to EN, especially in terms of protein delivery. This is important since some physicians treating critically ill patients may be reluctant to introduce EN in these patients or they simply keep them on PN.

Despite the technical advances in PN over the last decade, the early use of PN is still debatable [14]. EN has been deemed the optimal route for feeding over PN, although achieving current recommendations in caloric and protein targets with EN nutrition delivery in the critically ill may be difficult [2,3,4]. The lack of use of PN is quite surprising since patients who already have malnutrition on ICU admission would benefit from achieving nutritional targets as much as possible, and supplementary PN may be helpful in this objective [15]. Indeed, PN may be considered early in patients at risk of developing malnutrition when admitted to the ICU based on current recommendations [2,4]. Almost two-thirds of our patients had malnutrition on ICU admission; however, PN was used early without a higher incidence of complications (e.g., catheter-related complications), even when laboratory data were analyzed.

PN was indicated by the attending intensivist as an alternative route for nutrition delivery when EN was impossible or insufficient following current recommendations [16,17]. Gastrointestinal dysfunction secondary to severe illness (e.g., ileus, bowel fistula, abdominal occlusion, postoperative for major surgical procedures, sepsis, etc.) was the main reason for selecting PN in our patients. Gastrointestinal dysfunction in critically ill patients is common and has been associated with impaired outcomes [18,19]. As previously mentioned, frequently, EN cannot cover all nutritional needs and PN helps enable adequate energy and protein provision safely. Contemporary studies reported a mean protein delivery of between 0.8 and 1.1 g·Kg^−1^·day^−1^ [20,21,22,23], and a recent prospective multinational cohort study in patients with ICU stays ≥ 5 days found that their protein intake did not achieve current recommendations (i.e., 1.2–1.3 g·Kg^−1^·day^−1^) [24]. Indeed, we show a similar delivery of nutrition therapy, regardless of the route of administration.

A comparison was also made between the subgroups of patients treated exclusively with PN and those who also received additional EN during their ICU stay. Among the 186 patients who received nutritional treatment by PN, EN was further administered to 39.8% of them. Despite the observational nature of the study, a daily evaluation to replace PN with EN was performed by the attending intensivist. In the PN-EN subgroup, EN was instituted following the discontinuation of PN or via progressive switching from PN to EN. In 10 patients, however, EN was stopped because of EN-related complications, such as a high GRV, and a switch from PN to EN was not possible. The appearance of complications following EN in these patients probably represents the persistence of gastrointestinal dysfunction caused by the critical illness present on ICU admission.

We also describe three main patterns of administration to progress nutrition therapy from the parenteral to the enteral route within the PN-EN subgroup. Despite progressive switching from PN to EN seeming to be the most logical approach, the initiation of EN following the discontinuation of PN was seen in one-third (29.3%) of the PN-EN subgroup. In a recent survey of the transition from total PN to EN in critically ill patients involving the participation of 105 healthcare professionals (62% from ICU departments), 96% of participants reported that the objective of nutritional route transition was to ensure the tolerance of a particular percentage of caloric and protein requirements provided by EN [20]. In relation to the threshold of the theoretical EN requirements for PN discontinuation, 74.3% of participants were in a range between 60% and 75%. Also, there was great variability in the choice of the type of enteral diet (e.g., polymeric diets with and without fiber, oligomeric diet, and glutamine-enriched peptide-based diet), although, in daily practice, 94.3% of participants started enteral feeding with progressive volumes over a 72-h period. The study concluded that further research is required to define the best strategy for optimizing the transition from PN to EN in ICU patients [25].

In our population, PN provided adequate nutrition delivery, however, additional EN may optimize caloric and protein requirements. Differences were observed in the caloric and protein intake after the first and second weeks of ICU admission in favor of the PN-EN subgroup. Indeed, the adjusted multivariate analysis showed that a lower mean protein intake during nutrition therapy, shorter ICU stays, and less time on invasive mechanical ventilation when needed, were associated with the use of PN alone. These findings can be interpreted in different ways. Firstly, the PN subgroup of patients with PN alone represents a less severe subgroup since they had shorter stays and less time on invasive mechanical ventilation. Secondly, the PN-EN subgroup required longer ICU stays, which could represent greater disease severity. Finally, the PN-EN subgroup received a higher caloric and protein delivery, representing an optimization of nutrition therapy [9]. Optimal protein delivery in the ICU may afford different benefits in terms of outcomes: less time on mechanical ventilation, improvement in healing after surgery, lower incidence of acquired muscle weakness, and lower associated costs [22,26].

The present data reflect a real-world practice of nutrition therapy delivered to adult critically ill patients; nevertheless, the observational design, heterogeneity of participants, and lack of strong conclusions should be viewed as limitations of this study. On the other hand, the multicenter nature, the significant number of patients within our population, and the nutritional therapy follow-up until day 14 after ICU admission are among the strengths of this study. The sample size could be adequate for the entire ICU population, but it may be not optimal for subgroup analysis based on the number of patients included in the PN-EN subgroup. Thus, the subgroup analysis results should be considered with caution. To avoid the confounding influence of illness severity, nutritional status, length of nutrition therapy, and population heterogeneity, we accounted for potential confounders along with the finest statistical performance, which aimed to minimize their influence (described in the Methods section). It is important to remark that patients from the EN-PN subgroup represented a different subgroup of patients and the use of PN in them is related to gastro-intestinal dysfunction (i.e., a higher average of EN-related complications) [27]. Even though the results are debatable, they are clinically relevant and could be helpful in better identifying those patients requiring PN and with expected longer ICU admissions who would benefit from adding EN to PN.

## 5. Conclusions

In the present observational cohort study regarding artificial nutrition therapy during ICU admission, one-third of the study population required PN. The parenteral route may be safe and without a higher incidence of complications, even when administered early, compared to the enteral route. PN may also help provide adequate nutrition delivery; however, additional EN, when possible, may optimize calorie and protein requirements, especially in patients who cannot receive nutrition therapy by the enteral route and require longer ICU stays.

## Figures and Tables

**Table 1:** General characteristics, nutritional support, and outcomes of patients receiving parenteral nutrition.

		All Patients *n* = 186	Only PN *n* = 112	PN-EN *n* = 74	*p*-Value
Baseline characteristics and comorbidities					
Age, years, mean ± SD		64.32 ± 13.90	63.8 ± 14.53	65.09 ± 12.95	0.53
Sex, male patients, *n* (%)		124 (66.7)	68 (60.7)	56 (75.7)	0.05
Body mass index, kg/m^2^, mean (range)		26.8 (14.7–41.1)	27.3 (14.7–40.2)	26.3 (17.3–41.2)	0.24
Hypertension, *n* (%)		89 (47.8)	53 (47.3)	36 (48.6)	0.88
Diabetes mellitus, *n* (%)		47 (25.3)	27 (24.1)	20 (27.0)	0.73
Chronic obstructive pulmonary disease, *n* (%)		27 (14.5)	14 (12.5)	13 (17.6)	0.39
Acute myocardial infarction, *n* (%)		26 (14.0)	17 (15.2)	9 (12.2)	0.66
Chronic liver disease, *n* (%)		10 (5.4)	22 (19.6)	16 (21.6)	0.89
Chronic renal failure, *n* (%)		22 (11.8)	13 (11.6)	9 (12.2)	0.95
Immunosuppression, *n* (%)		23 (12.4)	13 (11.6)	10 (13.5)	0.81
Neoplasia, *n* (%)		65 (35.0)	43 (38.4)	22 (29.7)	0.27
Type of patient	Medical, *n* (%)	79 (42.3)	43 (39.4)	36 (48.6)	**0.02**
	Trauma, *n* (%)	8 (4.3)	2 (1.8)	6 (8.1)	
	Surgery, *n* (%)	99 (53.2)	67 (59.8)	32 (43.2)	
APACHE II, mean ± SD		19.78 ± 7.47	18.95 ± 6.98	21.05 ± 8.03	0.06
SAPS II, mean ± SD		49.80 ± 18.59	48.02 ± 19.38	52.44 ± 17.15	0.13
SOFA at ICU admission, mean ± SD		6.81 ± 4.04	6.14 ± 4.04	7.81 ± 3.85	**0.005**
Patient with malnutrition (based on SGA), *n* (%)		110 (59.1)	65 (58.0)	45 (60.8)	0.76
mNUTRIC score, mean ± SD		4.55 ± 1.94	4.39 ± 1.98	4.78 ± 1.85	0.18
Patient at risk based on mNUTRIC score, *n* (%)		90 (48.4)	52 (46.4)	38 (51.3)	0.76
Nutritional support					
Time of PN initiation, h, mean ± SD		38.34 ± 35.19	41.62 ± 38.5	33.36 ± 29.04	0.12
Early PN, <48 h, *n* (%)		140 (75.3)	82 (73.2)	58 (78.4)	0.49
Kcal/kg/day *, mean ± SD		19.94 ± 6.72	19.27 ± 7.24	20.96 ± 5.74	0.09
Protein, g/kg/day *, mean ± SD		1.01 ± 0.41	0.95 ± 0.43	1.17 ± 0.36	**0.03**
Outcomes					
Mechanical ventilation, *n* (%)		144 (77.4)	76 (67.9)	68 (91.9)	**0.001**
Mechanical ventilation, days, mean ± SD		12.23 ± 17.48	7.33 ± 11.24	17.71 ± 21.29	**<0.001**
Vasoactive drug support, *n* (%)		140 (75.3)	72 (64.3)	68 (91.9)	**<0.001**
Renal replacement therapy, *n* (%)		37 (19.9)	21 (18.7)	16 (21.62)	0.71
Respiratory tract infection, *n* (%)		56 (30.1)	35 (31.2)	21 (28.4)	0.74
Catheter-related infections, *n* (%)		15 (8.1)	12 (10.7)	3 (4.0)	0.08
ICU stay, days, mean ± SD		18.56 ± 12.61	11.51 ± 10.46	24.34 ± 24.63	**<0.001**
Hospital stay, days, mean ± SD		39.49 ± 31.36	32.56 ± 21.7	50.03 ± 45.78	**<0.001**
28-day mortality, *n* (%)		46 (24.7)	27 (24.1)	19 (25.7)	0.86

PN: parenteral nutrition; EN: enteral nutrition; SD: standard deviation; APACHE II: Acute Physiology and Chronic Health Disease Classification System II; SAPS: Simplified Acute Physiology Score; SOFA: Sequential Organ Failure Assessment; SGA: Subjective Global Assessment; mNUTRIC: modified Nutrition Risk in the Critically Il; and ICU: intensive care unit. Statistically significant *p*-values are written in bold. * During the entire administration of nutrition therapy or at least the first 14 days.

**Table 2:** General characteristics, nutritional support, and outcomes of patients receiving parenteral and enteral nutrition.

		Only PN *n* = 112	Only EN *n* = 400	EN-PN *n* = 43	*p*-Value **
Baseline characteristics and comorbidities					
Age, years, mean ± SD		63.8 ± 14.53	60.72 ± 15.45	60.23 ± 13.56	0.33
Sex, male patients, *n* (%)		68 (60.7)	267 (66.7%)	31 (72.1)	0.39
Body mass index, kg/m^2^, mean (range)		27.3 (14.7–40.2)	28.17 ± 6.32	27.12 ± 4.92	0.17
Hypertension, *n* (%)		53 (47.3)	170 (42.5)	17 (39.5)	0.24
Diabetes mellitus, *n* (%)		27 (24.1)	103 (25.7)	13 (30.2)	0.91
Chronic obstructive pulmonary disease, *n* (%)		14 (12.5)	72 (18.0)	9 (20.9)	0.55
Acute myocardial infarction, *n* (%)		17 (15.2)	59 (14.7)	7 (16.3)	0.89
Chronic liver disease, *n* (%)		22 (19.6)	22(5.5)	0	0.13
Chronic renal failure, *n* (%)		13 (11.6)	42 (10.5)	3 (7.0)	0.88
Immunosuppression, *n* (%)		13 (11.6)	42 (10.5)	6 (13.9)	0.86
Neoplasia, *n* (%)		43 (38.4)	61 (15.2)	8 (18.6)	**0.01**
Type of patient	Medical, *n* (%)	43 (39.4)	285 (71.2)	31 (72.1)	**0.001**
	Trauma, *n* (%)	2 (1.8)	59 (14.7)	4 (9.3)	
	Surgery, *n* (%)	67 (59.8)	56 (14.0)	8 (18.6)	
APACHE II, mean ± SD		18.95 ± 6.98	20.04 ± 7.85	22.49 ± 8.03	0.16
SAPS II, mean ± SD		48.02 ± 19.38	48.37 ± 17.41	51.03 ± 15.94	0.43
SOFA at ICU admission, mean ± SD		6.14 ± 4.04	7.09 ± 3.17	8.65 ± 3.54	**0.04**
Patient with malnutrition (based on SGA), *n* (%)		65 (58.0)	138 (34.5)	16 (37.2)	0.16
mNUTRIC score, mean ± SD		4.39 ± 1.98	3.96 ± 2.17	4.81 ± 2.17	0.18
Patient at risk based on mNUTRIC score, *n* (%)		52 (46.4)	165 (41.2)	27 (62.8)	0.76
Nutritional support					
Time of nutrition initiation, h, mean ± SD		41.62 ± 38.5	36.40 ± 31.31	44.15 ± 25.49	**0.02**
Early nutrition therapy, <48 h, *n* (%)		82 (73.2)	308 (77.0)	26 (60.5)	0.69
Kcal/kg/day *, mean ± SD		19.27 ± 7.24	14.50 ± 5.60	15.46 ± 5.31	**<0.001**
Protein, g/kg/day *, mean ± SD		0.95 ± 0.43	0.76 ± 0.34	0.83 ± 0.28	**0.03**
Outcomes					
Mechanical ventilation, *n* (%)		76 (67.9)	391 (97.7)	40 (93)	**0.007**
Mechanical ventilation, days, mean ± SD		7.33 ± 11.24	13.23 ± 13.94	21.50 ± 18.35	**0.03**
Vasoactive drug support, *n* (%)		72 (64.3)	296 (74.0)	32 (74.4)	**0.01**
Renal replacement therapy, *n* (%)		21 (18.7)	41 (10.2)	17 (39.5)	0.31
Respiratory tract infection, *n* (%)		35 (31.2)	102 (25.5)	8 (18.6)	0.64
Catheter-related infections, *n* (%)		12 (10.7)	26 (6.5)	2 (4.6)	0.08
ICU stay, days, mean ± SD		11.51 ± 10.46	18.58 ± 16.23	23.88 ± 19.50	**<0.001**
Hospital stay, days, mean ± SD		32.56 ± 21.7	34.60 ± 29.80	39.20 ± 28.15	**0.001**
28-day mortality, *n* (%)		27 (24.1)	99 (24.7)	16 (37.2)	0.86

PN: parenteral nutrition; EN: enteral nutrition; SD: standard deviation; APACHE II: Acute Physiology and Chronic Health Disease Classification System II; SAPS: Simplified Acute Physiology Score; SOFA: Sequential Organ Failure Assessment; SGA: Subjective Global Assessment; mNUTRIC: modified Nutrition Risk in the Critically Il; and ICU: intensive care unit. Statistically significant *p*-values are written in bold. * During the entire administration of nutrition therapy or at least the first 14 days. ** *p*-value of the comparison between only PN and only EN subgroups.

**Table 3:** Mean caloric and protein requirements of ICU patients receiving parenteral (PN) and enteral nutrition (EN) during their ICU stay.

Day	Kcal/kg/Day			Protein g/kg/Day		
	Only PN *n* = 112	Only EN *n* = 400	*p*-Value	Only PN *n* = 112	Only EN *n* = 400	*p*-Value
1	14.71 ± 8.58	6.86 ± 4.73	**<0.001**	0.73 ± 0.51	0.36 ± 0.27	**<0.001**
2	19.93 ± 8.37	12.97 ± 6.81	**<0.001**	0.99 ± 0.60	0.68 ± 0.38	**0.001**
3	20.05 ± 7.70	15.56 ± 7.36	**0.001**	1.00 ± 0.46	0.81 ± 0.43	0.07
4	20.18 ± 7.34	16.54 ± 7.38	**0.004**	1.03 ± 0.47	0.88 ± 0.42	0.13
5	20.27 ± 7.37	17.11 ± 7.40	0.08	1.03 ± 0.47	0.90 ± 0.42	0.17
6	20.10 ± 8.85	17.61 ± 7.45	0.09	1.03 ± 0.47	0.95 ± 0.43	0.25
7	20.16 ± 9.64	17.72 ± 7.71	0.08	1.04 ± 0.51	0.95 ± 0.42	0.24
Mean 1st week	13.62 ± 4.89	10.11 ± 4.37	**0.001**	0.94 ± 0.42	0.72 ± 0.33	**0.04**
8	21.02 ±10.10	18.13 ± 7.33	**0.01**	1.06 ± 0.49	0.96 ± 0.43	0.28
9	23.73 ± 8.04	18.20 ± 7.24	**0.001**	1.08 ± 0.54	0.98 ± 0.43	0.44
10	22.93 ± 9.48	18.44 ± 7.56	0.05	1.08 ± 0.60	0.98 ± 0.43	0.36
11	25.03 ± 8.22	19.36 ± 7.07	**0.001**	1.16 ± 0.60	1.04 ± 0.40	0.13
12	24.79 ± 8.94	19.53 ± 7.25	**0.001**	1.26 ± 0.53	1.04 ± 0.43	0.07
13	25.61 ± 6.90	19.75 ± 6.76	**0.001**	1.27 ± 0.50	1.03 ± 0.44	0.10
14	21.98 ± 7.92	20.50 ± 6.67	0.88	1.09 ± 0.54	1.05 ± 0.43	0.51
Mean 2nd week	19.27 ± 7.24	14.50 ± 5.60	**0.01**	0.95 ± 0.43	0.76 ± 0.34	0.06

PN: parenteral nutrition; EN: enteral nutrition. Statistically significant *p*-values are written in bold.

**Table 4:** Mean caloric and protein requirements during parenteral nutrition (PN) delivery.

Day	Kcal/kg/Day				Protein g/kg/Day			
	All Patients *n* = 186	Only PN *n* = 112	PN-EN *n* = 74	*p*-Value	All Patients *n* = 186	Only PN *n* = 112	PN-EN *n* = 74	*p*-Value
1	14.46 ± 8.37	14.71 ± 8.58	14.07 ± 8.07	0.60	0.72 ± 0.46	0.73 ± 0.51	0.72 ± 0.39	0.88
2	19.92 ± 8.09	19.93 ± 8.37	19.91 ± 7.71	0.98	0.99 ± 0.52	0.99 ± 0.60	0.99 ± 0.40	0.94
3	20.82 ± 7.58	20.05 ± 7.70	21.93 ± 7.32	0.10	1.04 ± 0.45	1.00 ± 0.46	1.11 ± 0.43	0.11
4	21.18 ± 7.76	20.18 ± 7.34	22.42 ± 8.14	0.06	1.08 ± 0.48	1.03 ± 0.47	1.14 ± 0.49	0.13
5	21.19 ± 7.99	20.27 ± 7.37	22.14 ± 8.54	0.15	1.10 ± 0.51	1.03 ± 0.47	1.17 ± 0.54	0.07
6	21.41 ± 8.69	20.10 ± 8.85	22.56 ± 8.45	0.10	1.12 ± 0.49	1.03 ± 0.47	1.20 ± 0.50	0.05
7	21.99 ± 8.97	20.16 ± 9.64	23.42 ± 8.19	0.05	1.15 ± 0.52	1.04 ± 0.51	1.23 ± 0.52	**0.04**
Mean 1st week	15.56 ± 6.57	13.62 ± 4.89	16.84 ± 7.21	**0.001**	0.99 ± 0.40	0.94 ± 0.42	1.07 ± 0.37	**0.04**
8	22.37 ± 9.04	21.02 ±10.10	23.14 ± 8.37	0.26	1.12 ± 0.52	1.06 ± 0.49	1.15 ± 0.54	0.38
9	23.31 ± 7.83	23.73 ± 8.04	23.10 ± 7.80	0.73	1.14 ± 0.51	1.08 ± 0.54	1.17 ± 0.50	0.44
10	23.26 ± 9.42	22.93 ± 9.48	23.42 ± 9.49	0.83	1.15 ± 0.54	1.08 ± 0.60	1.19 ± 0.50	0.38
11	21.48 ± 8.55	25.03 ± 8.22	19.99 ± 8.32	**0.030**	1.06 ± 0.52	1.16 ± 0.60	1.02 ± 0.48	0.33
12	23.40 ± 9.04	24.79 ± 8.94	22.76 ± 9.13	0.44	1.18 ± 0.51	1.26 ± 0.53	1.14 ± 0.50	0.44
13	23.91 ± 7.93	25.61 ± 6.90	23.20 ± 8.31	0.32	1.18 ± 0.50	1.27 ± 0.50	1.14 ± 0.50	0.40
14	23.36 ± 10.26	21.98 ± 7.92	23.97 ± 11.23	0.58	1.14 ± 0.60	1.09 ± 0.54	1.16 ± 0.63	0.71
Mean 2nd week	19.94 ± 6.72	19.27 ± 7.24	20.96 ± 5.74	0.09	1.01 ± 0.41	0.95 ± 0.43	1.08 ± 0.36	**0.03**

PN: parenteral nutrition; EN: enteral nutrition. Statistically significant *p*-values are written in bold.

**Table 5:** Results of multivariate analyses of factors associated with the use of parenteral nutrition (PN) only compared against the use of parenteral and enteral nutrition (PN-EN) during an ICU stay.

Variables	Hazard Ratio (95% Confidence Interval)	*p*-Value
Mean g of protein/kg/day	0.29 (0.18–0.47)	**0.001**
Mechanical ventilation	0.51 (0.26–1.29)	0.15
Days on mechanical ventilation	0.85 (0.81–0.89)	**0.001**
Vasoactive drug support	0.90 (0.89–1.87)	0.35
Mean length of ICU stay	0.96 (0.92–0.99)	**0.008**
Catheter-relation infections	3.91 (0.84–8.83)	0.115

Quality of the model: Akaike Information Criterion (AIC) = 196.03; Bayesian Information Criterion (BIC) = 221.84; and Pseudo R^2^ (McFadden) = 0.28. Statistically significant *p*-values are written in bold.

**Table 6:** Laboratory data of patients receiving parenteral nutrition (PN) admitted to the ICU.

		All Patients (*n* = 186)	PN Only (*n* = 112)	PN-EN (*n* = 74)	*p*-Value
Lipid profile					
Cholesterol, mean ± SD (mg/dL)	Day 1	99 ± 47	102 ± 49	96 ± 43	0.53
	Day 3	99 ± 38	99 ± 35	99 ± 44	0.97
	Day 7	113 ± 42	107 ± 31	118 ± 48	0.26
	ICU discharge	123 ± 48	119 ± 38	130 ± 61	0.28
High cholesterol levels (>200 mg/dL), *n* (%)		8 (4.3)	1 (0.9)	7 (9.5)	0.32
Triglycerides, mean ± SD (mg/dL)	Day 1	148 ± 114	134 ± 100	168 ± 129	0.14
	Day 3	186 ± 110	173 ± 97	203 ± 126	0.20
	Day 7	187 ± 87	178 ± 79	194 ± 94	0.44
	ICU discharge	185 ± 101	190 ± 86	176 ± 124	0.54
Hypertriglyceridemia (>350 mg/dL), *n* (%)		16 (8.6)	6 (5.4)	10 (13.5)	0.24
Liver parameters					
Bilirubin, mean ± SD (mg/dL)	Day 1	2.47 ± 1.55	1.31 ± 1.25	3.62 ± 1.92	0.11
	Day 3	2.46 ± 1.36	1.54 ± 1.20	3.42 ± 1.59	0.32
	Day 7	2.23 ± 1.36	2.74 ± 1.32	1.64 ± 1.39	0.87
	ICU discharge	2.51 ± 1.22	2.67 ± 1.16	2.25 ± 1.31	0.72
High bilirubin levels (>1.23 mg/dL), *n* (%)		77 (41.4)	46 (41.1)	31 (41.9)	0.70
High transaminases (AST or ALT > 40 IU/L), *n* (%)		108 (58.1)	58 (51.8)	50 (67.6)	0.68
High ALP levels (>129 UI/L), *n* (%)		91 (48.9)	51 (45.5)	40 (54.0)	**0.05**
High GGT levels (>67 IU/L), *n* (%)		132 (71.0)	75 (67.0)	57 (77.0)	0.80
Liver dysfunction, *n* (%)		75 (40.3)	48 (42.9)	27 (36.5)	0.41
Blood proteins and C-reactive protein					
Prealbumin, mean ± SD (mg/L)	Day 1	117.5 ± 70.7	115.9 ± 72.9	119.9 ± 68.1	0.80
	Day 3	108.4 ± 64.5	106.1 ± 68.7	112.9 ± 55.9	0.64
	Day 7	144.7 ± 68.7	149.1 ± 69.8	140.9 ± 68.8	0.65
	ICU discharge	164.9 ± 77.8	171.8 ± 81.2	150.7 ± 69.8	0.27
Low prealbumin levels, <200 mg/L, *n* (%)		117 (62.9)	72 (64.3)	45 (60.8)	0.43
Albumin, ± SD (g/L)	Day 1	2.54 ± 0.65	2.51 ± 0.62	2.58 ± 0.71	0.50
	Day 3	2.39 ± 0.52	2.40 ± 0.52	2.37 ± 0.51	0.74
	Day 7	2.42 ± 0.57	2.45 ± 0.55	2.39 ± 0.59	0.62
	ICU discharge	2.53 ± 0.63	2.52 ± 0.64	2.54 ± 0.62	0.87
Low albumin levels, <30 g/L, *n* (%)		175 (94.1)	107 (95.5)	68 (91.9)	0.89
C-reactive protein, mean ± SD (mg/L)	Day 1	173.6 ± 144.2	171.2 ± 133.5	177.3 ± 160.1	0.79
	Day 3	152.8 ± 128.6	168.8 ± 135.6	128.09 ± 113.9	0.07
	Day 7	144.7 ± 90.6	120.5 ± 97.8	129.62 ± 81.3	0.10
	ICU discharge	104.2 ± 109.9	119.4 ± 123.7	76.07 ± 71.2	**0.03**

SD: standard deviation; ALT: alanine aminotransferase; AST: aspartate aminotransferase; ALP: alkaline phosphatase; and GGT: gamma-glutamyltransferase. Statistically significant *p*-values are written in bold.

## Data Availability

The data presented in this study are available via contact with the corresponding author of the present manuscript. Data requests should be evaluated by the local ethics committee in order to agree with legal requirements.

## Supplementary Materials

The following supporting information can be downloaded at: https://www.mdpi.com/article/10.3390/nu15214665/s1, Table S1: Mechanical and infectious complications related to Parenteral Nutrition and central venous catheter evaluated during the study; Table S2: Mean caloric and protein requirements during parenteral nutrition (PN) delivery during ICU stay. Comparison between EN-PN subgroup and only PN subgroup (A), and EN-PN subgroup and PN-EN subgroup; Table S3: Laboratory data of the patients receiving parenteral nutrition admitted to the ICU: lipid profile (A), liver parameters (B), and blood proteins & C-reactive protein (C).

## Appendix A

The following list comprises the members of the ENPIC (study of the Evaluation of Nutritional Practices In the Critical care patient) collaboration, and should be acknowledged as collaborating authors: Lluís Servia-Goixart, Javier Trujillano-Cabello, Joan Escobar-Ortiz, Neus Montserrat-Ortiz, and Amalia Zapata-Rojas (H. Universitari Arnau de Vilanova, Lleida); Teodoro Grau-Carmona, Iris Bautista-Redondo, Ana Cruz-Ramos, Laura Diaz-Castellanos, Miriam Morales-Cifuentes, Montserrat Plaza-Bono, Juan Carlos Montejo-Gonzalez, Susana Temprano-Vazquez, and Veronica Arjona-Diaz (H. Universitario 12 de Octubre, Madrid); Carlos Garcia-Fuentes, Carolina Mudarra-Reche, and Maria Orejana-Martin (H. Universitario 12 de Octubre, UCI de Trauma y Emergencia, Madrid); Juan Carlos Lopez-Delgado, Africa Lores-Obradors, Laura Anguela-Calvet, Gloria Muñoz-Del Río, Pamela Alejandra Revelo-Esquibel, Henry Alanez-Saavedra, Pau Serra-Paya, Stephani Maria Luna-Solis, and Alvaro Salinas-Canovas (H. Universitari Bellvitge, L’Hospitalet del Llobregat, Barcelona); Fernando De Frutos-Seminario and Oriol Rodriguez-Queralto (H. Universitari Bellvitge, Unidad Coronaria, L’Hospitalet del Llobregat, Barcelona); Carlos Gonzalez-Iglesias and Monica Zamora-Elson (H. de Barbastro, Barbastro, Huesca); Eugenia de la Fuente-O’Connor (H. Universitario Infanta Sofía, San Sebastian de los Reyes, Madrid); Carlos Seron-Arbeloa and Nestor Bueno-Vidales (H. San Jorge, Huesca); Rayden Iglesias-Rodriguez (H. General de Granollers, Barcelona); Ana Martin-Luengo (H. Rio Hortega, Valladolid); Angel Sanchez-Miralles, Enrique Marmol-Peis, Miriam Ruiz-Miralles, Maria Gonzalez-Sanz, and Arantzazu Server-Martinez, (H. Sant Joan d’ Alacant, Alicante); Belen Vila-Garcia (H. Infanta Cristina de Parla, Madrid); Carol Lorencio-Cardenas (H. Universitari Josep Trueta, Girona); Laura Macaya-Redin, Raquel Flecha-Viguera, and Sara Aldunate-Calvo (H. de Navarra, Pamplona); Jose Luis Flordelis-Lasierra, Irene Jimenez-del Rio, Jose Ramon Mampaso-Recio, and Jose Manuel Rodriguez-Roldan (H. Universitario Severo Ochoa, Leganés, Madrid); Rosa Gastaldo-Simeon and Josefina Gimenez-Castellanos (H. Manacor, Mallorca); Juan Francisco Fernandez-Ortega, Juan F Martinez-Carmona, Esther Lopez-Luque, and Ane Ortega-Ordiales (H. Regional Universitario Carlos Haya, Málaga); Monica Crespo-Gomez, Victor Ramirez-Montero, Esther Lopez-García, and Arturo Navarro-Lacalle (H. Universitari Doctor Peset, Valencia); Pilar Martinez-Garcia and Maria Inmaculada Dominguez-Fernandez (H. Universitario de Puerto Real, Cadiz); Paula Vera-Artazcoz, Marta Izura-Gomez, and Susana Hernandez-Duran (Hospital de la Santa Creu i Sant Pau, Barcelona); Mª Luisa Bordeje-Laguna, Esther Mor-Marco, Yaiza Rovira-Valles, and Viridiana Philibert (H. Universitari Germans Trias i Pujol, Badalona, Barcelona); Maravillas de las Nieves Alcazar-Espin and Aurea Higon-Cañigral (H. G. Universitario Morales Meseguer, Murcia); Enrique Calvo-Herranz, Diego Manzano-Moratinos (H. Universitario de Getafe, Madrid); Esther Portugal-Rodriguez, David Andaluz-Ojeda, Laura Parra-Morais, Rafael Citores-Gonzalez, Maria Teresa Garcia-Gonzalez, and Gloria Renedo Sanchez-Giron (H. Clínico Universitario de Valladolid; Elisabeth Navas-Moya, Carles Ferrer-Pereto, Cristina Lluch-Candal, Jessica Ruiz-Izquierdo, and Silvia Castor-Bekari (H. Mutua Terrassa, Barcelona); Cristina Leon-Cinto (H. Royo Villanova, Zaragoza); Itziar Martinez de Lagran and Juan Carlos Yebenes-Reyes (H. de Mataro, Barcelona); Beatriz Nieto-Martino and Clara Vaquerizo-Alonso (H. Universitario de Fuenlabrada, Madrid); Susana Almanza-Lopez, Sonia Perez-Quesada, and Jose Luis Anton-Pascual (H.G. Universitario de Alicante); Judith Marin-Corral, Maite Sistachs-Baquedano, Maria Hacer-Puig, and Marina Picornell-Noguera (H. del Mar, Barcelona); Lidon Mateu-Campos, Clara Martinez-Valero, and Andrea Ortiz-Suñer (H.G. Universitario de Castellón); Beatriz Llorente-Ruiz, María Cristina Martinez-Diaz, Maria Trascasa- Muñoz De La Peña, and Diego Anibal Rodriguez-Serrano (H. Príncipe de Asturias, Alcalá de Henares, Madrid); Leticia Fernandez-Salvatierra, Mireia Barcelo-Castello, and Paula Millan-Taratiel (H. Clínico Lozano Blesa, Zaragoza); Antonio Tejada-Artigas, Ines Martinez-Arroyo, Pilar Araujo-Aguilar, Maria Fuster-Cabre, Laura Andres-Gines, and Sonia Soldado-Olmo (H. Miguel Servet, Zaragoza); Eva Mª Menor-Fernandez, Lucas Lage-Cendon, and Alberto Touceda-Bravo (H. Álvaro Cunqueiro, Vigo); Laura Sanchez-Ales and Laura Almorin-Gonzalvez (H. Plató, Barcelona); Maria Gero-Escapa, Esther Martinez-Barrio, and Sergio Ossa-Echeverri (H. Universitario de Burgos); and Diana Monge-Donaire (Hospital Virgen de la Concha, Zamora).
