# Supplementary material for: Parenteral Nutrition: Current Use, Complications, and Nutrition Delivery in Critically Ill Patients
Source: Nutrients. 2023 Nov 3;15(21):4665. doi: 10.3390/nu15214665 (PMC10649219; doi:10.3390/nu15214665)
Supplement: Supplementary file 1 [file nutrients-15-04665-s001.zip › nutrients-2639227-supplementary.pdf]

Table S1. Mechanical and infectious complications related to Parenteral Nutrition and central venous catheter evaluated during the study.

**Mechanical complications**	
Related with catheter insertion	***Clinical considerations for diagnosis***
Arterial puncture	Symptomatic
Arterial laceration	If surgical repair is required
Gas embolism	If there is clinical manifestation
Catheter embolism	By radiological confirmation
Brachial plexus injury	If there are clinical symptoms
Pneumothorax / Pneumomediastinum	With compatible clinical and radiology
Hemothorax / Hemomediastinum	
Hydrothorax	lipid-free PN in pleural space
Chylothorax	Lesion of the thoracic duct
Cardiac lesion	Clinic & diagnosis of pericardial tamponade
Arrhythmias (with the removal of the catheter)	Significant hemodynamic instability
Arterio-venous fistulae	Confirmed echography diagnosis
Related with catheter maintenance	***Clinical considerations for diagnosis***
Vein thrombosis	Confirmed echography diagnosis
Thrombophlebitis	Clinical diagnosis
**Infectious complications**	
Catheter infection	Catheter-related infection was defined according to:<br>• Catheter tip colonization: significant growth of a microorganism (>15 colony-forming units) from the catheter tip.<br>• Catheter-related local infection: any sign of local infection (induration, erythema, heat, pain, purulent drainage) and catheter tip colonization criteria.<br>• Catheter-related bloodstream infection: a positive blood culture obtained from a peripheral vein, and signs of systemic infection (fever, chills, and/or hypotension), with no apparent source of bacteremia except the catheter, and catheter tip colonization with the same organism.

Table S2. Mean caloric and protein requirements during parenteral nutrition (PN) delivery during ICU stay. Comparison between EN-PN subgroup and only PN subgroup (A), and EN-PN subgroup and PN-EN subgroup.

A	Kcal/kg/day			Protein g/kg/day		
Day	Only PN *n* = 112	EN-PN *n* = 43	*p-Value*	Only PN *n* = 112	EN-PN *n* = 43	*p-Value*
1	14.71 ± 8.58	6.52 ± 4.55	***0.01***	0.73 ± 0.51	0.36 ± 0.27	***0.01***
2	19.93 ± 8.37	12.07 ± 7.56	***0.01***	0.99 ± 0.60	0.64 ± 0.39	***0.03***
3	20.05 ± 7.70	16.31 ± 8.23	***0.04***	1.00 ± 0.46	0.89 ± 0.48	*0.09*
4	20.18 ± 7.34	15.89 ± 7.28	***0.04***	1.03 ± 0.47	0.85 ± 0.38	*0.23*
5	20.27 ± 7.37	17.65 ± 7.08	*0.12*	1.03 ± 0.47	0.91 ± 0.39	*0.15*
6	20.10 ± 8.85	18.61 ± 7.98	*0.09*	1.03 ± 0.47	0.98 ± 0.42	*0.35*
7	20.16 ± 9.64	16.66 ± 8.66	*0.08*	1.04 ± 0.51	0.95 ± 0.46	*0.40*
Mean 1st week	13.62 ± 4.89	10.62 ± 5.09	***0.01***	0.94 ± 0.42	0.79 ± 0.27	***0.04***
8	21.02 ±10.10	16.27 ± 9.74	***0.03***	1.06 ± 0.49	0.89 ± 0.46	*0.18*
9	23.73 ± 8.04	16.22 ± 10.03	***0.02***	1.08 ± 0.54	0.89 ± 0.45	*0.50*
10	22.93 ± 9.48	17.32 ± 11.04	*0.05*	1.08 ± 0.60	0.90 ± 0.58	*0.65*
11	25.03 ± 8.22	17.50 ± 8.95	***0.01***	1.16 ± 0.60	0.87 ± 0.51	*0.28*
12	24.79 ± 8.94	19.28 ± 6.15	***0.01***	1.26 ± 0.53	1.01 ± 0.35	*0.12*
13	25.61 ± 6.90	19.69 ± 6.82	***0.01***	1.27 ± 0.50	1.04 ± 0.46	*0.23*
14	21.98 ± 7.92	20.87 ± 8.60	*0.78*	1.09 ± 0.54	1.10 ± 0.41	*0.65*
Mean 2nd week	19.27 ± 7.24	15.46 ± 5.31	***0.03***	0.95 ± 0.43	0.36 ± 0.27	*0.07*

B	Kcal/kg/day			Protein g/kg/day		
Day	PN-EN<br>n = 74	EN-PN<br>*n* = 43	*p-Value*	PN-EN<br>n = 74	EN-PN<br>*n* = 43	*p-Value*
1	14.07 ± 8.07	6.52 ± 4.55	***0.01***	0.72 ± 0.39	0.36 ± 0.27	***0.01***
2	19.91 ± 7.71	12.07 ± 7.56	***0.01***	0.99 ± 0.40	0.64 ± 0.39	***0.04***
3	21.93 ± 7.32	16.31 ± 8.23	*0.05*	1.11 ± 0.43	0.89 ± 0.48	*0.12*
4	22.42 ± 8.14	15.89 ± 7.28	*0.06*	1.14 ± 0.49	0.85 ± 0.38	*0.18*
5	22.14 ± 8.54	17.65 ± 7.08	*0.18*	1.17 ± 0.54	0.91 ± 0.39	*0.12*
6	22.56 ± 8.45	18.61 ± 7.98	*0.10*	1.20 ± 0.50	0.98 ± 0.42	*0.30*
7	23.42 ± 8.19	16.66 ± 8.66	*0.08*	1.23 ± 0.52	0.95 ± 0.46	*0.21*
Mean 1st week	16.84 ± 7.21	10.62 ± 5.09	***0.03***	1.07 ± 0.37	0.79 ± 0.27	***0.04***
8	23.14 ± 8.37	16.27 ± 9.74	*0.07*	1.15 ± 0.54	0.89 ± 0.46	*0.22*
9	23.10 ± 7.80	16.22 ± 10.03	***0.04***	1.17 ± 0.50	0.89 ± 0.45	*0.45*
10	23.42 ± 9.49	17.32 ± 11.04	*0.09*	1.19 ± 0.50	0.90 ± 0.58	*0.65*
11	19.99 ± 8.32	17.50 ± 8.95	***0.04***	1.02 ± 0.48	0.87 ± 0.51	*0.68*
12	22.76 ± 9.13	19.28 ± 6.15	*0.06*	1.14 ± 0.50	1.01 ± 0.35	*0.22*
13	23.20 ± 8.31	19.69 ± 6.82	*0.09*	1.14 ± 0.50	1.04 ± 0.46	*0.53*
14	23.97 ±11.23	20.87 ± 8.60	*0.82*	1.16 ± 0.63	1.10 ± 0.41	*0.75*
Mean 2nd week	20.96 ± 5.74	15.46 ± 5.31	***0.04***	1.08 ± 0.36	0.83 ± 0.28	***0.04***

PN: parenteral nutrition; EN: enteral nutrition. Statistically significant *p*-values are written in bold.

Table S3. Laboratory data of the patients receiving parenteral nutrition admitted to the ICU: lipid profile (A), liver parameters (B), and blood proteins & C-reactive protein (C).

A		All patients (n=186)	PN only (n=112)	PN-EN (n=74)	*p-Values*
Triglycerides, mean ± SD (mg/dL)	Day 1	148.59 ± 114.32	134.12 ± 100.57	168.70 ± 129.68	*0.14*
	Day 3	186.19 ± 110.83	173.79 ± 97.43	203.95 ± 126.91	*0.20*
	Day 7	187.16 ± 87.84	178.10 ± 79.14	194.36 ± 94.59	*0.44*
	ICU discharge	185.32 ± 101.40	190.34 ± 86.20	176.54 ± 124.69	*0.54*
Hypertriglyceridemia (> 350 mg/dL), n (%)		16 (8.6)	6 (5.4)	10 (13.5)	*0.24*
Cholesterol, mean ± SD (mg/dL)	Day 1	99.93 ± 47.11	102.36 ± 49.81	96.39 ± 43.23	*0.53*
	Day 3	99.21 ± 38.99	99.10 ± 35.90	99.41 ± 44.19	*0.97*
	Day 7	113.80 ± 42.13	107.33 ± 31.21	118.92 ± 48.89	*0.26*
	ICU discharge	123.24 ± 48.13	119.24 ± 38.94	130.73 ± 61.84	*0.28*
High cholesterol levels (> 200 mg/dL), n (%)		8 (4.3)	1 (0.9)	7 (9.5)	*0.32*
HDL, mean ± SD (mg/dL)	Day 1	24.57 ± 19.17	24.79 ± 21.34	24.23 ± 15.75	*0.90*
	Day 3	34.09 ± 54.84	40.34 ± 65.01	22.96 ± 26.94	*0.22*
	Day 7	25.32 ± 32.52	22.41 ± 24.79	27.42 ± 37.43	*0.59*
	ICU discharge	24.13 ± 12.58	24.60 ± 14.50	23.40 ± 9.13	*0.74*
Low HDL levels (< 40 mg/dL), n (%)		92 (49.5)	52 (46.4)	40 (54.0)	*0.27*
LDL, mean ± SD (mg/dL)	Day 1	50.33 ± 41.60	49.50 ± 46.13	51.48 ± 35.17	*0.85*
	Day 3	156.53 ± 437.63	176.59 ± 528.97	119.29 ± 176.40	*0.63*
	Day 7	71.08 ± 64.57	77.05 ± 86.66	66.27 ± 40.12	*0.57*
	ICU discharge	70.96 ± 40.33	64.00 ± 32.56	82.31 ± 49.42	*0.12*
High LDL levels (>190 mg/dL), n (%)		12 (6.4)	8 (7.1)	4 (5.4)	*0.56*

SD: standard deviation; HDL: high-density lipoprotein cholesterol; LDL: low-density lipoprotein cholesterol.

B		All patients (n=186)	PN only (n=112)	PN-EN (n=74)	*p-Values*
ALT/ GPT, mean ± SD (IU/L)	Day 1	276.7 ± 101.4	325.9 ± 109.5	177.0 ± 88.9	*0.63*
	Day 3	610.5 ± 183.3	540.5 ± 137.6	705.2 ± 254.6	*0.25*
	Day 7	228.1 ± 88.1	145.1 ± 66.5	283.4 ± 107.7	*0.36*
	ICU discharge	694.3 ± 154.1	852.4 ± 173.4	289.8 ± 122.1	*0.67*
AST/ GOT, mean ± SD (IU/L)	Day 1	351.4 ± 130.5	403.5 ± 127.2	264.2 ± 135.2	*0.89*
	Day 3	935.1 ± 232.9	374.4 ± 104.1	443.6 ± 142.4	***0.03***
	Day 7	123.1 ± 67.6	59.3 ± 49.6	157.1 ± 82.7	*0.19*
	ICU discharge	833.7 ± 183.2	964.7 ± 179.5	584.4 ± 189.1	*0.95*
High transaminases (AST or ALT>40 IU/L), n (%)		108 (58.1)	58 (51.8)	50 (67.6)	*0.68*
Bilirubin, mean ± SD (mg/dL)	Day 1	2.47 ± 1.55	1.31 ± 1.25	3.62 ± 1.92	*0.11*
	Day 3	2.46 ± 1.36	1.54 ± 1.20	3.42 ± 1.59	*0.32*
	Day 7	2.23 ± 1.36	2.74 ± 1.32	1.64 ± 1.39	*0.87*
	ICU discharge	2.51 ± 1.22	2.67 ± 1.16	2.25 ± 1.31	*0.72*
High bilirubin levels (> 1.23 mg/dL), n (%)		77 (41.4)	46 (41.1)	31 (41.9)	*0.70*
ALP, mean ± SD (IU/L)	Day 1	119.8 ± 116.3	110.2 ± 94.4	133.3 ± 74.2	*0.26*
	Day 3	114.6 ± 81.6	115.5 ± 75.8	113.3 ± 90.4	*0.88*
	Day 7	167.6 ± 99.3	149.7 ± 62.7	180.8 ± 81.1	*0.16*
	ICU discharge	192.4 ± 154.1	173.2 ± 116.4	222.9 ± 97.7	*0.10*
High ALP levels (> 129 UI/L), n (%)		91 (48.9)	51 (45.5)	40 (54.0)	***0.05***
GGT, mean ± SD (IU/L)	Day 1	210.5 ± 142.1	174.3 ± 131.2	252.6 ± 157.1	*0.47*
	Day 3	139.1 ± 119.8	147.1 ±111.7	165.5 ± 131.1	*0.44*
	Day 7	264.1 ± 256.2	263.1 ± 130.8	250.9 ± 201.1	*0.83*
	ICU discharge	316.3 ± 271.9	372.2 ± 292.4	238.8 ± 195.1	*0.39*
High GGT levels (> 67 IU/L), n (%)		132 (71.0)	75 (67.0)	57 (77.0)	*0.80*
Liver dysfunction, n (%)		75 (40.3)	48 (42.9)	27 (36.5)	*0.41*

SD: standard deviation; ALT: alanine aminotransferase; GPT: glutamate-pyruvate transaminase; AST: aspartate aminotransferase; GOT: glutamic oxaloacetic transaminase; ALP: alkaline phosphatase; GGT: gamma-glutamyltransferase.

C		All patients (n=186)	PN only (n=112)	PN-EN (n=74)	*p-Values*
Prealbumin, mean ± SD (mg/L)	Day 1	117.5 ± 70.7	115.9 ± 72.9	119.9 ± 68.1	*0.80*
	Day 3	108.4 ± 64.5	106.1 ± 68.7	112.9 ± 55.9	*0.64*
	Day 7	144.7 ± 68.7	149.1 ± 69.8	140.9 ± 68.8	*0.65*
	ICU discharge	164.9 ± 77.8	171.8 ± 81.2	150.7 ± 69.8	*0.27*
Low prealbumin levels, < 200 mg/L, n (%)		117 (62.9)	72 (64.3)	45 (60.8)	*0.43*
Albumin, ± SD (g/L)	Day 1	2.54 ± 0.65	2.51 ± 0.62	2.58 ± 0.71	*0.50*
	Day 3	2.39 ± 0.52	2.40 ± 0.52	2.37 ± 0.51	*0.74*
	Day 7	2.42 ± 0.57	2.45 ± 0.55	2.39 ± 0.59	*0.62*
	ICU discharge	2.53 ± 0.63	2.52 ± 0.64	2.54 ± 0.62	*0.87*
Low albumin levels, < 30 g/L, n (%)		175 (94.1)	107 (95.5)	68 (91.9)	*0.89*
C-reactive protein, mean ± SD (mg/L)	Day 1	173.6 ± 144.2	171.2 ± 133.5	177.3 ± 160.1	*0.79*
	Day 3	152.8 ± 128.6	168.8 ± 135.6	128.09 ± 113.9	*0.07*
	Day 7	144.7 ± 90.6	120.5 ± 97.8	129.62 ± 81.3	*0.10*
	ICU discharge	104.2 ± 109.9	119.4 ± 123.7	76.07 ± 71.2	***0.03***

SD: standard deviation.
